# A Prospective Cross-Sectional Study on the Vitamin D Status of Neonates and the Impact of Neonates’ Standard Vitamin D Supplementation on Neonatal Morbidities

**DOI:** 10.3390/children11050543

**Published:** 2024-05-01

**Authors:** Ezgi Yangin Ergon, Bayram Ali Dorum, Hanife Gul Balki, Derya Bako, Senem Alkan Ozdemir

**Affiliations:** 1 Clinic of Neonatology, T.C. Ministry of Health, Van Provincial Health Directorate, H.S.U. Van Training and Research Hospital, Van 65300, Turkey; bayramali.dorum@saglik.gov.tr; 2 Clinic of Pediatrics, T.C. Ministry of Health, Van Provincial Health Directorate, H.S.U. Van Training and Research Hospital, Van 65300, Turkey; hanifegul.balki@saglik.gov.tr; 3 Clinic of Pediatric Radiology, T.C. Ministry of Health, Van Provincial Health Directorate, H.S.U. Van Training and Research Hospital, Van 65300, Turkey; derya.bakokeskin@saglik.gov.tr; 4 Clinic of Neonatology, T.C. Ministry of Health, Izmir Provincial Health Directorate, H.S.U. Dr Behcet Uz Pediatric Diseases and Surgery Training and Research Hospital, Izmir 35210, Turkey; senem.alkanozdemir@sbu.edu.tr

**Keywords:** morbidity, newborn, supplementation, vitamin D

## Abstract

(1) Background: This study aimed to determine whether standard-dose vitamin D supplementation could provide adequate levels in exclusively breastfed infants born with different cord 25(OH)D levels and to evaluate related neonatal morbidities. (2) Methods: A prospective cross-sectional study was designed in term infants. Three groups were formed based on cord 25(OH)D levels; Group1 Deficiency:25(OH)D < 12 ng/mL, Group2 Insufficiency:25(OH)D = 12–19 ng/mL, and Group3 Optimum:25(OH)D = 20–100 ng/mL. Cord and 1st month 25(OH)D levels, after receiving standard-dose vitamin D prophylaxis, neonatal outcomes and anthropometric measurements were compared. (3) Results: The study involved 86 infants. Group1 Deficiency had also significantly lower 25(OH)D levels at 1st month compared to the others (*p* < 0.001). There was a significant positive correlation between cord and 1st month 25(OH)D levels (r = 0.78, *p* < 0.001). Despite the fact that the mother’s age and clothing style were similar, Group1 Deficiency mothers had higher parity numbers and used less vitamin D during pregnancy (*p* = 0.03, *p* = 0.04). Neonatal outcomes analysis revealed that newborns in Group1 Deficiency experienced more respiratory distress, transient tachypnea, and early-onset sepsis, as well as more and longer hospital stays in the first-month of life (*p* < 0.05). (4) Conclusions: Infants with low cord 25(OH)D levels had also lower 25(OH)D values in the 1st month of life and experienced higher rates of neonatal morbidities. Given its numerous biological activities and impact on neonatal morbidities, determining an individualized dose of vitamin D supplementation may be more accurate than using the standard approach.

## 1. Introduction

Today, vitamin D insufficiency is becoming more widespread as a silent pandemic, despite all the advancements in diet and health technologies. Vitamin D has many biological activities, including maintaining calcium homeostasis, protecting bone health, and regulating bone metabolism. It contributes to immune system, neurological system, and cardiovascular system-regulating processes. In adults, vitamin D has been linked to a number of illnesses, including rheumatoid arthritis, diabetes, depression, schizophrenia, Parkinson’s disease, Alzheimer’s disease, liver, kidney, metabolic, and oncological diseases [1,2].

In contrast to other vitamins, only a minor portion of vitamin D is found in diets, the majority of vitamin D is produced spontaneously in the skin through the conversion of the steroid precursor 7-dehydrocholesterol to ultraviolet B (UVB) radiation. Sunlight-induced UVB rays that reach the skin are influenced by a number of parameters, including age, body weight, sun exposure time, skin hyperpigmentation, season, and latitude [3,4].

Based on the biological unity of mother and child, attention has been drawn to vitamin D inadequacy as a prevalent issue during the past 20 years, and perinatal vitamin D deficiency has acquired significance in this context. The most significant risk factor for vitamin D deficiency in newborns and early childhood is inadequate maternal vitamin D. Pregnancy is a crucial time, and the effects of vitamin D on the fetus are lifelong due to its extra-bone effect [5]. In light of all of this, the Ministry of Health in our country proposed in 2011 that all pregnant women receive a single dose of 1200 IU of vitamin D per day, beginning after the second trimester of pregnancy and continuing for a total of 12 months until the sixth month after birth [6]. Pregnant women do not, however, adhere to this preventive strategy very often [7]. Furthermore, lack of sun exposure and breastfeeding without nutritional supplements are two major factors contributing to low serum 25-hydroxy vitamin D [25(OH)D] concentrations in healthy children [8,9,10]. Breastfeeding alone provides 11–38 IU of 25(OH)D per day [9,10]. If supplementation is not provided, infants who are breastfed exclusively run the danger of having insufficient serum 25(OH)D concentrations [9].

Vitamin D supplementation is the most commonly recommended method for establishing adequate reserves of vitamin D in infants. A serum 25(OH)D concentration of 20 mg/dL in newborns to one-year-olds is thought to be supported by vitamin D supplementation of 200–400 IU per day beginning in the first month of life and continuing until it is obtained from other foods, according to randomized controlled studies [9,11,12,13,14,15]. Similar preventive vitamin D levels are used for newborns worldwide, despite the knowledge that numerous factors affect a baby’s ability to synthesize vitamin D and that insufficiency can have numerous neonatal and long-term outcomes.

The purpose of the study was to evaluate the relationship between vitamin D deficiency and neonatal morbidities as well as whether routine vitamin D supplementation could provide adequate levels in exclusively breastfed newborns born with varying cord 25(OH)D levels.

## 2. Materials and Methods

A prospective cross-sectional investigation was carried out at the Health Science University (HSU) Van Training and Research Hospital. This hospital, located in Turkey’s Eastern Anatolia region with unusually high birth rates, includes a level 4 neonatal critical care facility with 80 incubators. There are approximately 2500 admissions per year. The yearly average temperature in this city is 9 °C. There are roughly 140 sunny days every year, with the coldest month’s average temperature being −3.5 °C and the warmest month’s average temperature being 22 °C [16].

### 2.1. Ethics and Consent

The study was approved by the institutional review board of the HSU Van Training and Research Hospital Clinical Research Ethics Committee and strictly followed the institution’s ethical guidelines (approval number 2019/01). Informed consent was obtained from the participants’ legal guardians.

### 2.2. Study Design

**Figure 1:**
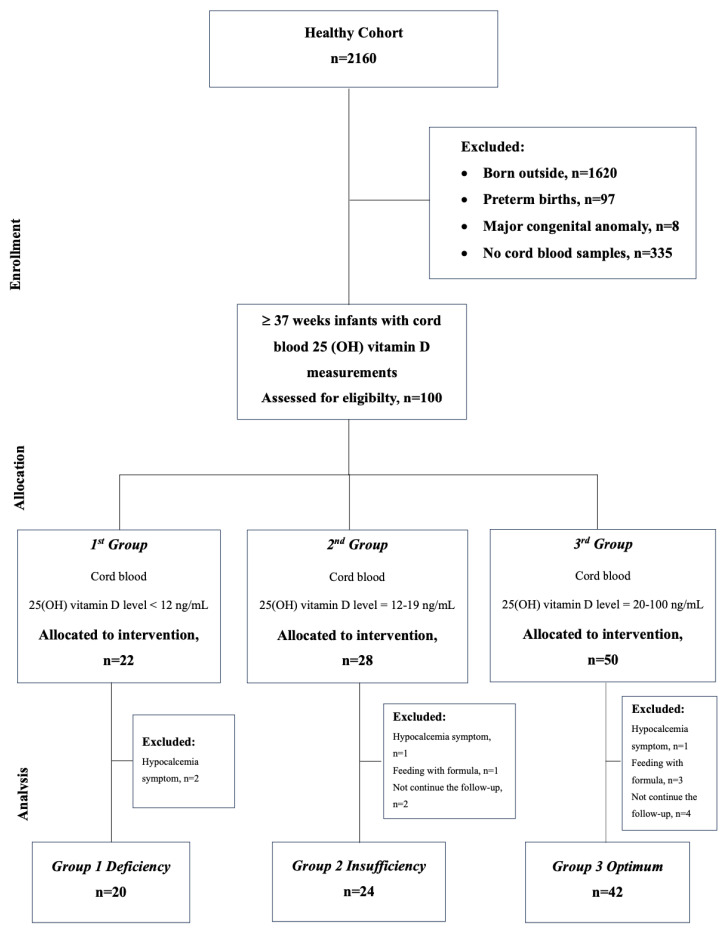
Flow chart for the selection of eligible infants in the study.

The study included term infants (GA ≥ 37 weeks) born during the same season. Infants with major congenital anomalies or whose cord blood samples could not be taken and for whom family written consent could not be obtained were not included in the study. Infants who required formula in addition to breast milk, did not take routine 400 IU vitamin D supplements (just vitamin D drops, not multivitamins such as A,C, and D), had hypocalcemia or hypomagnesemia during follow-up, or missed follow-up appointments were all excluded from the study. The flow chart of selected eligible infants in the study is presented in Figure 1.

### 2.3. Procedure

Following delivery, three milliliters of cord blood were collected from infants who met the study criteria to determine serum concentrations of 25(OH)D, calcium (Ca), magnesium (Mg), phosphorus (P), albumin, parathormone (PTH), and alkaline phosphatase (ALP). Blood samples were collected and sent to the laboratory in accordance with the cold chain protocol. Calcium, P, Mg, albumin, and ALP levels in serum samples were measured using enzymatic, kinetic, and end-point methods on a Beckman Coulter AU2700 analyser with original reagents (Brea, CA, USA). The electrochemiluminescence method was used on an analyzer to measure serum PTH levels (Beckman Coulter DXI Brea, CA, USA), as well as 25(OH)D (Roche Diagnostics Cobas e401 GMBH Mannheim, Germany). The global consensus defines vitamin D deficiency and insufficiency in children as <12 and 12–20 ng/mL, respectively, in the prevention and treatment of nutrition-related rickets [17]. Based on this consensus, three groups were formed based on cord 25(OH) D vitamin levels; Group 1: 25(OH)D < 12 ng/mL (Deficient); Group 2: 25(OH)D = 12–19 ng/mL (Insufficient); and Group 3: 25(OH)D = 20–100 ng/mL (Optimum).

### 2.4. Data Collection

The electronic medical record system and the patient sheets were used to gather information on the clinical characteristics of the infants, such as gender, type of birth, head circumference (HC), GA, and birth weight (BW), as well as the demographic and characteristic features of the mothers, such as age, parity, weight gain, clothing style, and calcium and vitamin D supplementation during pregnancy. Infants were assessed in the outpatient clinic for a month with weekly checks (postnatal 3rd day, 1st week, 2nd week, 3rd week, and 1st month) and an analysis of dietary patterns, length of sun exposure, necessity for hospitalization (hospitalization on the day of life or duration of hospital stay), and the reasons for hospitalization (sepsis, respiratory distress, and hyperbilirubinemia). Anthropometric measurements, hip and kidney ultrasonography (US), and laboratory markers obtained from cord blood were repeated at the first month follow-up. The study used the reference values indicated below: Ca (8.0 to 11.0) (mg/dL), Mg (1.6 to 2.2) (mg/dL), P (4.0 to 8.0) (mg/dL), albumin (3.5 to 5.5) (g/dL), PTH (10.0 to 65.0) (pg/mL), and ALP (48.0 to 406.0) (U/L) [18,19,20].

### 2.5. Sample Size

The amount of standard vitamin D supplementation in 80 newborns was to be monitored. In terms of neonatal morbidity, patients with normal and low vitamin D levels (deficient and insufficient) were divided into two groups of at least 40 infants each. All newborns who met the research criteria and whose families agreed to participate were included in the study, along with all infants born in the same season at a single facility, until the target number was reached.

### 2.6. Statistical Analysis

The variable analysis was carried out using the program SPSS 25.0 (IBM Corporation, Armonk, New York, USA). Normally distributed numerical data was analyzed using parametric methods, whereas non-normally distributed and categorical numerical data was analyzed using nonparametric techniques. The Kolmogorov-Smirnov test was used to evaluate normalization (*p* > 0.05). Descriptive statistics were provided in percentages, mean ± SD, median, and interquartile range (25–75 percentiles) based on the normal data distribution. The relationships between categorical variables within three groups were compared using chi-square and one-way ANNOVA tests. Bonferroni correction was made in ANNOVA post hoc analyses. Pearson’s correlation coefficient was used to determine the relationship between 25(OH) D levels in cord serum and the first month. A *p*-value of less than 0.05 indicated a significant difference between categories.

## 3. Results

### 3.1. Demographical Characteristics

Table 1 summarizes the demographic data for the enrolled infants as well as the characteristics of their mothers.

In our research, despite similar mother ages and outfits, deficiency group mothers utilized less vitamin D supplement throughout pregnancy and had greater parity numbers than the other groups, which led to a statistically significant difference [Parity mean ± SDS, *p* = 0.03, Vitamin D supplementation n (%), *p* = 0.04, respectively].

### 3.2. Primary Outcomes

**Figure 2:**
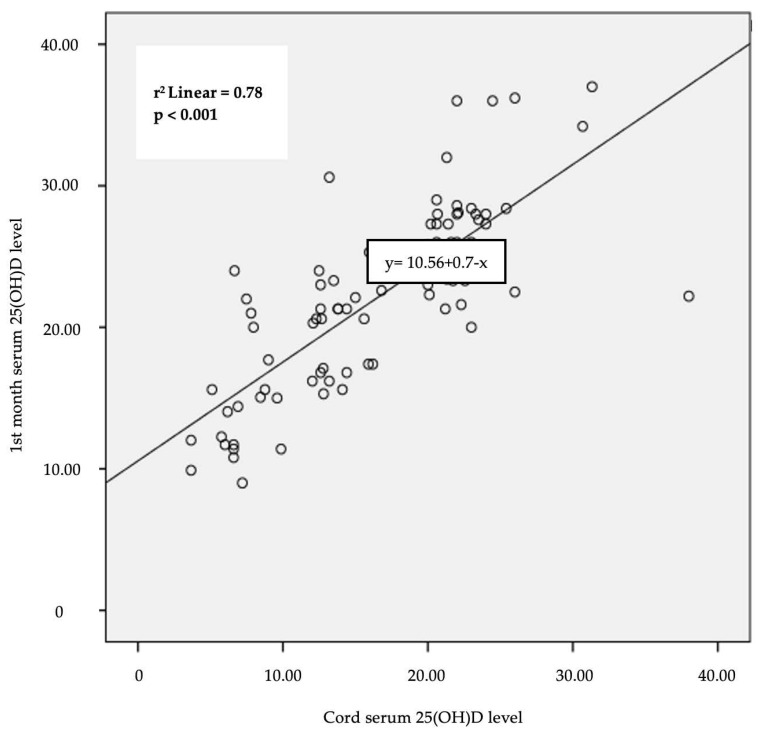
Correlation between cord serum 25(OH)D and 1st month serum 25(OH)D levels (Pearson’s correlation coefficient).

The infants in the deficiency group had significantly lower 25(OH)D levels in the first month when we examined the cord, first month 25(OH) D levels, and other laboratory results in all infants who were given standard vitamin D supplements for one month [First month 25(OH)D (ng/mL) mean ± SDS, *p* < 0.001]. There was a significant positive correlation between cord and 1st month serum 25(OH) D levels [r = 0.78, *p* < 0.001] (Figure 2).

Although the infants in the Group 1 Deficiency had statistically significantly lower levels of Ca, P, and albumin in their cord blood, this difference disappeared by the first month [Cord Ca(mg/dL) mean ± SDS, *p* < 0.001; Cord P (mg/dL) mean ± SDS, *p* = 0.03; cord albumin (g/dL) mean ± SDS, *p* = 0.03, respectively]. In the Group 3 Optimum, the 1st month PTH level was found to be the lowest, creating a statistically significant difference compared to the other groups [1st month PTH (pg/mL) mean ± SDS, *p* = 0.05] (Table 2).

### 3.3. Secondary Outcomes

An analysis of neonatal outcomes showed that throughout the first month of life, infants in the Group 1 Deficiency had more respiratory distress, transient tachypnea, early-onset sepsis (EOS), hospitalizations, and longer hospital stays than newborns in the other groups [Respiratory distress n (%), *p* = 0.01; transient tachypnea n (%), *p* = 0.03; EOS n (%), *p* = 0.05; hospitalization n (%), *p* = 0.008; hospitalization time (/days) mean ± SDS, *p* < 0.001, respectively] (Table 3).

In the first month of evaluation, anthropometric measurements were similar between the groups (*p* > 0.05). One newborn (2%) in Group 3 Optimum and two (10%) in Group 1 Deficiency had type 2A hips on hip US, while no infant’s renal US showed any stones [21] (Table 3).

There were notable differences between the deficient and insufficient groups in terms of parity number of mothers, infants’ cord, first month 25(OH)D levels, and respiratory distress (*p* = 0.03, *p* < 0.001, *p* < 0.001, and *p* = 0.03 respectively). However, only the infants’ levels of cord, first month 25(OH)D, and PTH differed substantially between the inadequate and normal groups (*p* < 0.001, *p* < 0.001, and *p* = 0.05, respectively). Furthermore, useage of vitamin D supplement during pregnancy, infants’ cord 25(OH)D, calcium, phosphate, albumin, first-month 25(OH)D levels, length of hospital stay, early-onset sepsis, and respiratory distress were all observed to differ statistically significantly between the deficient vs. optimal group (*p* = 0.05, *p* < 0.001, *p* < 0.001, *p* < 0.001, *p* = 0.02, *p* = 0.04, *p* < 0.001, *p* = 0.04 and *p* = 0.01, respectively) (Table 1, Table 2 and Table 3).

## 4. Discussion

In our study, we assessed vitamin D levels at the end of the first month as well as neonatal outcomes in breastfed infants who were divided into three groups based on the cord vitamin D values and received standard vitamin D supplements. Upon comparing babies with low cord 25(OH)D levels to those with appropriate levels, it was shown that the low 25(OH)D levels persisted at the end of the first month with standard-dose vitamin D supplementation, and these infants exhibited higher morbidity.

It was once widely believed that UVB radiation was sufficient to prevent vitamin D deficiency, but this belief was disproved in low-latitude countries where vitamin D fortification has been in place for years. Nevertheless, vitamin D deficiency is a worldwide serious public health concern, affecting people of all ages [22]. In Palacios et al.‘s meta-analysis, which evaluated a period of the last decade, they found that the prevalence of vitamin D deficiency was 24–44% in developed countries and 67–72% in developing countries [22]. Vitamin D deficiency is also common in pregnant women and infants, with prevalence rates ranging from 4% to 60% and from 3% to 86%, respectively, based on studies from different countries [22,23]. Eighteen meta-analyses focused on the preventive effect of vitamin D preparations on the development of obstetric and pediatric pathology. Vitamin D deficiency has been linked to a higher risk of preterm labor, particularly in multiple pregnancies, spontaneous abortion, preeclampsia, pregnancy anemia, postpartum depression, and early childhood autism spectrum disorders. A reduced risk of low birth weight and gestational diabetes is associated with high vitamin D levels [24]. Vitamin D deficiency during pregnancy has an impact on maternal and neonatal health [25]. Because of these dangers, prophylactic use of vitamin D is recommended for pregnant women in our country [6]. Nevertheless, pregnant women’s compliance with this prophylactic approach is low, and a study conducted in our country in 2022 showed that only 27.1% of pregnant women used vitamin D supplements during pregnancy [7]. Our findings also supported this; while the rate of mothers using vitamin D supplements during pregnancy was found to be low at 26.7% in the whole study group, this rate was found to be dramatically lower, up to 5%, and the number of parities was higher in mothers of infants with low cord vitamin D.

Since vitamin D insufficiency can have long-term repercussions on the mother and growing fetus, it is important to investigate what can be done during pregnancy to alleviate deficiency or minimize risks.

While it is known that maternal serum and cord blood 25(OH)D levels are highly correlated, both maternal serum and cord blood 25(OH)D increase after maternal vitamin D supplementation [26,27]. In addition to the correlation between maternal serum and cord 25(OH)D levels, studies in the literature have demonstrated a positive correlation between the levels of 25(OH)D in the venous blood of six-month-old infants and cord blood [7,28,29]. As reported in the literature, our study also revealed a significant correlation between cord and 1st month serum 25(OH)D levels. Infants with low cord 25(OH)D levels had also low 1st month 25(OH)D levels. In this study, postnatal standard 400 IU vitamin D supplements were given to all breastfed infants as well as those born with low cord vitamin D due to mothers who did not take adequate vitamin D supplements during pregnancy.

The recommended daily intake of vitamin D supplements for infants was 200 IU in 2003; however, in 2008, the American Academy of Pediatrics revised this recommendation to 400 IU [9]. The Canadian Academy of Pediatrics advised giving breastfed infants 400 IU of vitamin D in the summer and 800 IU in the winter (11). All children in the United Kingdom up to the age of three should take vitamin D supplements, and individuals who are at-risk (i.e., those who are Asian in origin) should receive support for five years [15]. Bulgaria recommends 800 IU of vitamin D, while Romania only recommends 400 IU [30,31]. The recommended vitamin D prophylactic dose for all infants under one year of age is 400 IU of vitamin D per day, in accordance with the health policy that has been in place in Turkey for the past two decades [32].

Dermal synthesis of vitamin D depends on the amount of UVB reaching the earth, the amount of ozone, seasons, time of day, closed clothing style, air pollution, latitude, skin pigmentation, weather conditions, excess body weight, duration of sun exposure, and the use of sunscreens. While immigration, global population growth, and skin pigmentation vary widely, determining a single dose of vitamin D supplements for a country or society raises the question of whether it is enough to prevent deficiency in the vitamin, which has many vital roles other than calcium metabolism [33,34,35]. Moreover, the complexity of this scenario is increased by the fact that different people respond differently to vitamin D supplemantation, and that individual responses are influenced by epigenetic characteristics [36].

Even infants born with extremely low birth weights can absorb and metabolize vitamin D, and vitamin D supplements can improve calcium absorption within four weeks [37]. The concentration of vitamin D and its metabolites in human milk does not correspond to 25(OH)D serum levels in breastfed infants. While there is a correlation between the content of vitamin D and the mother’s consumption, human milk is insufficient for providing vitamin D to breastfed infants. Instead, the infants get most of their vitamin D via endogenous synthesis upon exposure to sunshine or from supplements. There is an age-dependent increase in vitamin D absorption. In neonates, forms of the vitamin taken orally reach equivalence in their capacity to increase circulation vitamin D levels at a postnatal age of about 89 days [38]. Preterm and term infants born with vitamin D deficiency are more likely to experience respiratory distress syndrome, neonatal transient tachypnea (TTN), acute lower respiratory tract infection, and early and late neonatal sepsis (LOS) than infants with optimal vitamin D levels, according to published research on the effects of vitamin D on the respiratory and immune systems [39,40,41,42,43]. As mentioned in the literature, our study also revealed that infants with low cord vitamin D levels had higher rates of respiratory distress from neonatal transient tachypnea and EOS, as well as longer hospital stays both in terms of frequency and duration. These infants, who are asymptomatic in terms of calcium metabolism, have low cord vitamin D levels and are at high risk of neonatal morbidity due to the deficiency of vitamin D, which is necessary for many biological processes.

Many earlier studies defined vitamin D deficiency as a 25(OH)D level < 20 ng/mL [34]. Nevertheless, global consensus recommendations on the prevention and treatment of nutritional rickets agree that vitamin D insufficiency is 12–20 ng/mL and vitamin D deficiency is less than 12 ng/mL [17]. Regarding the vitamin D level for rickets prevention, there is agreement in today’s literature; however, there is uncertainty over whether the level is adequate for other bioactivities or what level lowers the risk of newborn morbidity.

Anthropometric measurements of newborn infants are one of many aspects of vitamin D and neonatal outcomes that have been studied in the literature. Several studies in the literature have either confirmed or refuted the association between anthropometric measurements and vitamin D insufficiency [44,45]. Lykkedegn et al. found some intriguing findings, including a U-shaped link between the weight of newborns at birth and the concentration of vitamin D in cord blood, and a significant increase in weight was observed when concentration values went beyond 24 ng/mL [46]. The vitamin D content in the cord was not found to be correlated with birth weight or first-month anthropometric parameters in our study. A larger case series could clarify this matter.

Our study was limited to infants born during a season and took place in a single region in the same climate, with infants of mothers wearing similar clothing. It ensured that many factors influencing vitamin D levels were comparable, allowing for a more accurate assessment of the effects of prophylactic vitamin D supplementation on the newborn baby’s vitamin D level. In addition to its strengths, our study has some limitations. Firstly, the study was limited to a single center. However, it could be expanded to include several centers. Secondly, a larger patient series could be examined, and thirdly, the infants’ long-term outcomes could be investigated. Lastly, based on the mother’s vitamin D usage during pregnancy, the correlation between the infants’ cord and first month vitamin D levels may be assessed, and neonatal morbidities could also be examined from this perspective.

Additionally, calculation the vitamin D concentration in breast milk, evaluating the correlation between the cord and first month vitamin D levels of infants comparing the mothers’ vitamin D levels during pregnancy are all very important and distinct aspects that may be encountered in various future studies. Our study will form the basis and shed light on these different studies in the future.

## 5. Conclusions

Finally, infants with low cord 25(OH)D levels had lower 25(OH)D values in the first month of life than infants with optimal cord 25(OH)D values. These infants showed no symptoms of calcium metabolism, but they had higher rates of newborn morbidities. Personalized dosage determination may be preferable over standard protocol since vitamin D has a wide variety of biological activities beyond calcium metabolism and is useful in lowering newborn morbidities.

## Figures and Tables

**Table 1:** Demographic Data of The Enrolled Infants and Characteristics of Their Mothers.

	Deficiency Group (*n* = 20) Group 1	Insufficiency Group (*n* = 24) Group 2	Optimum Group (*n* = 42) Group 3	*p* Value ^a^	*p* Value ^b^		
					1^®^2	2^®^3	1^®^3
**Mother**							
**Age (year)** (mean ± SD)	26 ± 7	26 ± 5	26 ± 5	0.96			
**Parity** (mean ± SD)	2.45 ± 1.14	1.79 ± 0.77	1.95 ± 0.69	0.03	0.03	1.00	0.09
**Weight gain during pregnancy (kg)** (mean ± SD)	14.85 ± 2.90	15.64 ± 3.63	15.12 ± 3.56	0.72			
**Calcium supplement** (n(%))	1 (5)	4 (16.6)	11 (26.1)	0.13			
**Vitamin D supplement** (n(%))	1 (5)	8 (33.3)	14 (33.3)	0.04	0.10	1.00	0.05
**Clothing style** (n(%)) (Modest dress & Scarf)	19 (95)	20 (83.3)	32 (76.1)	0.18			
***Newborn***							
**Gender (boy)** (n(%))	10 (50)	13 (54.1)	26 (61.9)	0.64			
**GA (week)** (mean ± SD)	38.8 ± 1.2	38.1 ± 2.6	38.6 ± 1.8	0.22			
**Delivery *(vaginal)*** (n(%))	12 (60)	11 (45.8)	28 (66.6)	0.25			
**BW (g)** (mean ± SD)	3300 ± 400	3200 ± 400	3300 ± 350	0.39			
**BL (cm)** (mean ± SD)	49.15 ± 1.38	49.71 ± 1.53	49.73 ± 1.63	0.29			
**HC (cm)** (mean ± SD)	34.11 ± 0.98	34.24 ± 1.20	34.27 ± 0.91	0.91			
**SGA** (n(%))	1 (5)	0	1 (2.3)	0.19			
**Sun exposure time** (n(%))	9 (45)	11 (45)	23 (54.7)	0.31			

GA: Gestational age, BW: Birth weight, BL: Birth length, HC: Head circumferance, SGA: Small for GA; mean ± SD: mean and standard deviation; n: number of cases; Sun exposure time: Time from 10.00 a.m. to 03.00 p.m. duration: > 15 min frequency: > 3/week, ^a^ chi-square and one-way ANNOVA tests, ^b^ ANNOVA posthoc test, Bonferroni.

**Table 2:** Evaluation of Infants’ 1st Month Cord 25 (OH)D Levels and Other Laboratory Results.

	Deficiency Group (*n* = 20) Group 1	Insufficiency Group (*n* = 24) Group 2	Optimum Group (*n* = 42) Group 3	*p* Value ^a^	*p* Value ^b^		
					1^®^2	2^®^3	1^®^3
**25(OH) vitamin D (ng/mL) (mean ± SD)**							
Cord blood	7 ± 1.7	14 ± 1.4	23 ± 3.5	<0.001	<0.001	<0.001	<0.001
1st month	15 ± 4.2	20 ± 3.6	27 ± 4.2	<0.001	<0.001	<0.001	<0.001
**Ca (mg/dL)** (mean ± SD)							
Cord blood	8.6 ± 0.5	8.8 ± 0.3	9 ± 0.4	<0.001	0.25	0.11	<0.001
1st month	8.9 ± 0.4	8.9 ± 0.3	9.1 ± 0.5	0.19			
**Mg (mg/dL)** (mean ± SD)							
Cord blood	1.98 ± 0.20	1.98 ± 0.15	2.01 ± 0.25	0.79			
1st month	2.06 ± 0.17	1.98 ± 0.11	2.05 ± 0.21	0.30			
**P (mg/dL)** (mean ± SD)							
Cord blood	4.80 ± 0.49	5.15 ± 0.54	5.21 ± 0.62	0.03	0.13	1.00	0.02
1st month	4.77 ± 0.43	4.93 ± 0.42	5.03 ± 0.57	0.16			
**Albumin (g/dL)** (mean ± SD)							
Cord blood	3.62 ± 0.33	3.69 ± 0.26	3.83 ± 0.32	0.03	1.00	0.24	0.04
1st month	3.68 ± 0.27	3.71 ± 0.19	3.74 ± 0.21	0.57			
**PTH (pg/mL)** (mean ± SD)							
Cord blood	44 ± 26	43 ± 24	42 ± 16	0.11			
1st month	39 ± 22	45 ± 20	34 ± 13	0.05	0.77	0.05	0.95
**ALP (IU/L)** (mean ± SD)							
Cord blood	176 ± 40	182 ± 45	181 ± 39	0.87			
1st month	180 ± 29	188 ± 41	187 ± 43	0.73			

Ca: Calcium, Mg: Magnesium, P: Phosphate, PTH: Parathormone, ALP: Alcaline Phosphatase; mean ± SD: mean and standard deviation, ^a^ one-way ANNOVA test, ^b^ ANNOVA posthoc test, Bonferroni.

**Table 3:** Short-Term Outcomes of Infants According to Cord Blood 25(OH)D Levels.

	Deficiency Group (*n* = 20) Group 1	Insufficiency Group (*n* = 24) Group 2	Optimum Group (*n* = 42) Group 3	*p* Value ^a^	*p* Value ^b^		
					1^®^2	2^®^3	1^®^3
**Hospitalization (n(%))**	14 (70)	9 (38)	12 (29)	0.008	0.26	1.00	0.47
Day on Hospitalization (mean ± SD)	4.71 ± 5.33	7.45 ± 6.12	5.01 ± 4.93	0.42			
Hospitalization time (/days) (mean ± SD)	7.14 ± 1.83	4.72 ± 1.73	3.58 ± 2.10	<0.001	0.62	0.11	<0.001
**Sepsis** (n(%))	10 (50)	5 (21)	3 (7)	0.06			
**Clinical sepsis** (n(%))	7 (35)	5 (21)	2 (5)	0.24			
**Culture proven sepsis** (n(%))	3 (15)	0	1 (2)	0.21			
**EOS** (n(%))	5 (25)	2 (8)	0	0.05	0.59	0.77	0.04
**LOS** (n(%))	5 (25)	3 (13)	3 (7)	0.81			
**Hyperbilirubinemia requiring phototherapy** (n(%))	2 (10)	5 (21)	6 (14)	0.11			
**Respiratory distress** (n(%))	12 (60)	4 (17)	4 (10)	0.01	0.03	1.00	0.01
**TTN** (n(%))	8 (40)	2 (8)	2 (4)	0.03	0.11	1.00	0.08
**Congenital pneumonia** (n(%))	3 (15)	2 (8)	2 (5)	0.11			
**Pneumonia** (n(%))	1 (5)	0	0	0.29			
**Hypocalcemia** (n(%))	0	0	0	NS			
**Stones on renal US** (n(%))	0	0	0	NS			
**Hip US pathology** (n(%))	2 (10)	0	1 (2)	0.17			
**1st month W (g)** (mean ± SD)	4018.55 ± 408.21	3971.20 ± 386.13	4112.98 ± 590.24	0.27			
**1st month L (cm)** (mean ± SD)	52.85 ± 1.68	53.52 ± 1.53	53.83 ± 1.67	0.07			
**1st month HC (cm)** (mean ± SD)	37.02 ± 1.05	37.44 ± 1.31	37.69 ± 1.09	0.13			

EOS: Early onset sepsis, LOS: Late onset sepsis, TTN: Transient tachypnea, US: Ultrasound, W: Weight, L: Length, HC: Head circumference; mean ± SD: mean and standard deviation; n: number of cases, ^a^ chi-square and one-way ANNOVA tests, ^b^ ANNOVA posthoc test, Bonferroni.

## Data Availability

Data is contained within the article.
